# Supplementary material for: DC3 is a method for deconvolution and coupled clustering from bulk and single-cell genomics data
Source: Nat Commun. 2019 Oct 10;10:4613. doi: 10.1038/s41467-019-12547-1 (PMC6787340; doi:10.1038/s41467-019-12547-1)
Supplement: Supplementary file 1 — Supplementary Information [file 41467_2019_12547_MOESM1_ESM.pdf]

DC3: Deconvolution and coupled clustering from bulk and single cell genomics data

Zeng *et al*.

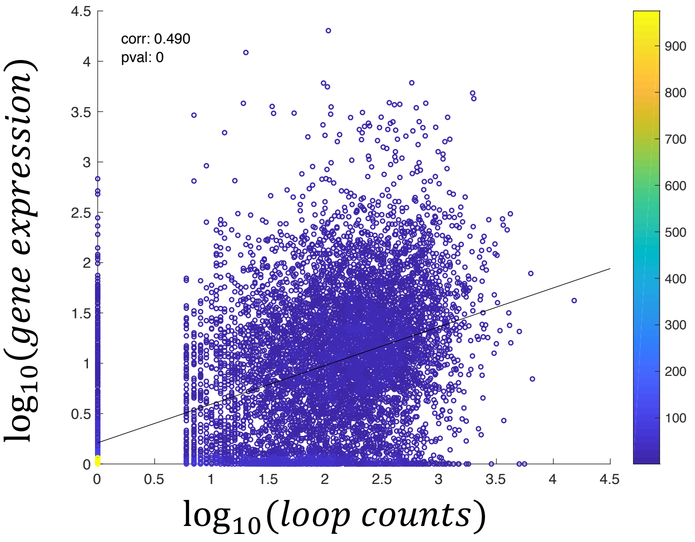

Supplementary Figure 1. The scatter plot between HiChIP loop counts and gene expression values in K562, the PCC score is 0.490. Source data is available upon request.

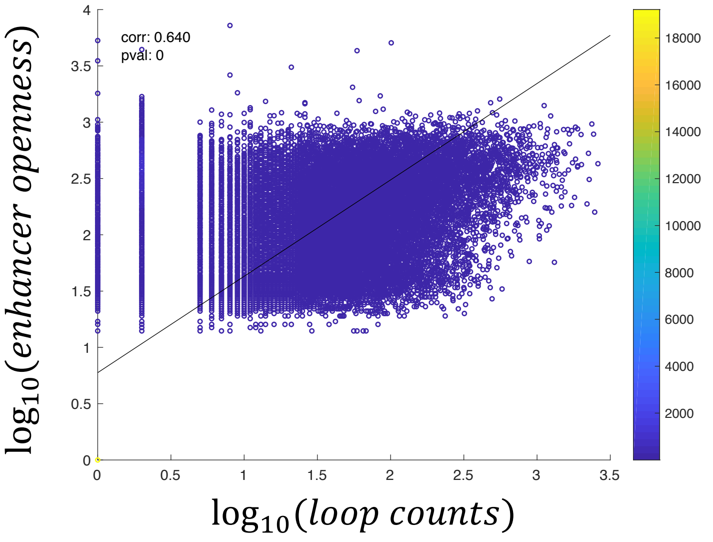

Supplementary Figure 2. The scatter plot between HiChIP loop counts and enhancer openness in K562, the PCC score is 0.640. Source data is available upon request.

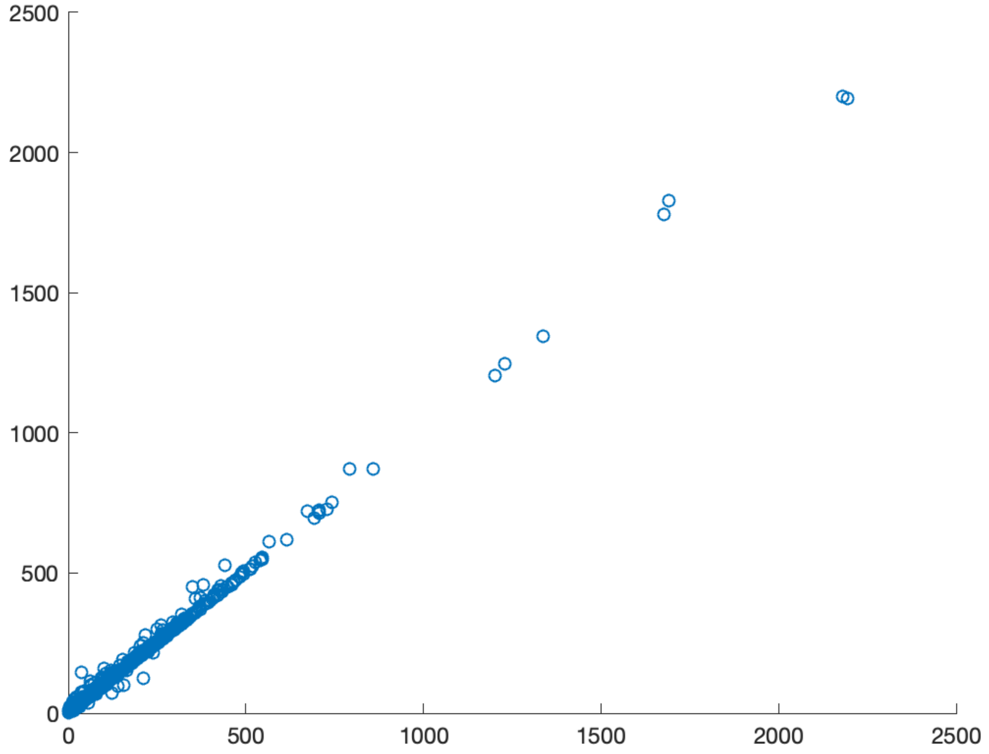

Supplementary Figure 3. The comparison results for the two methods to obtain subpopulation HiChIP profiles. The x-axis indicates the PCC for Poisson-based method and the y-axis indicates the deconvolution results for the simple method. Source data is available upon request.

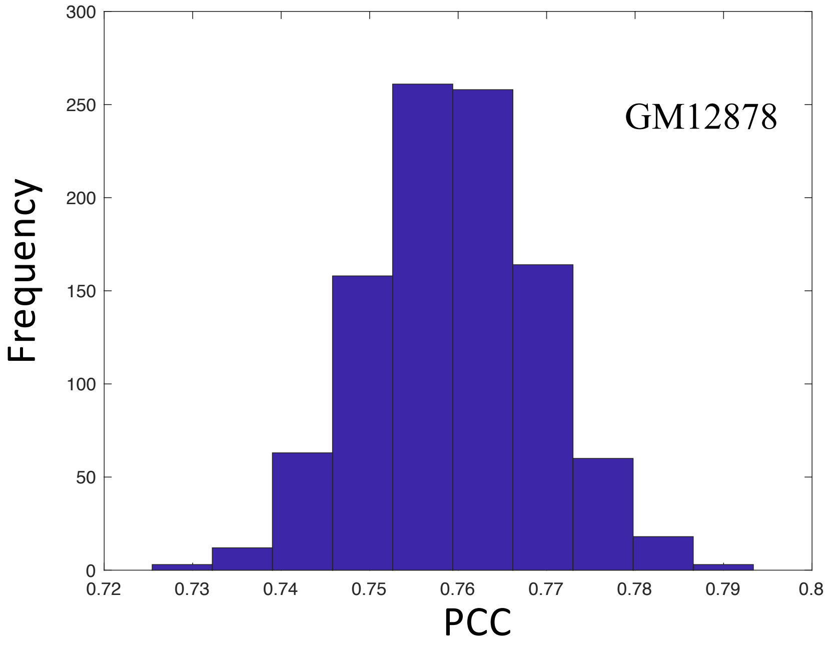

Supplementary Figure 4. The distribution of 1000 random HiChIP deconvolution results in GM12878. Source data is available upon request.

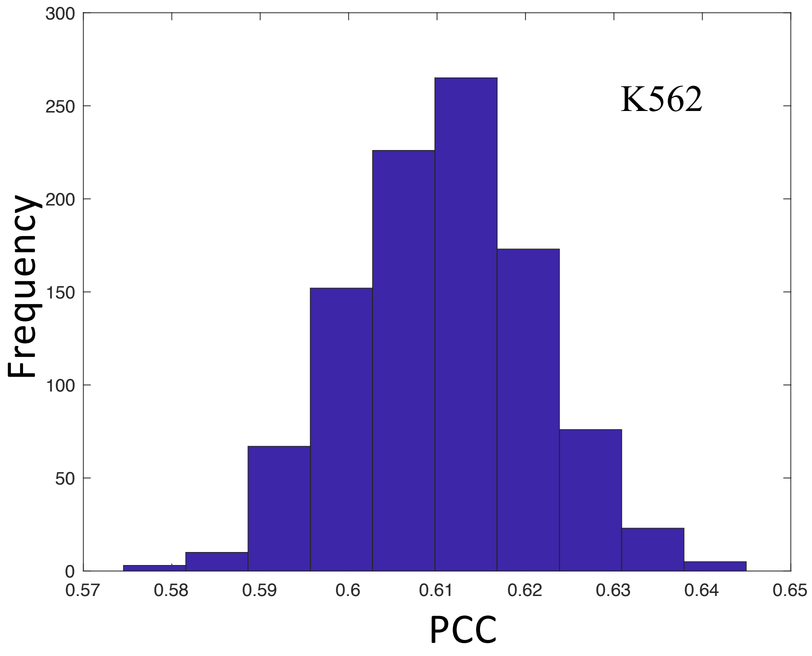

Supplementary Figure 5. The distribution of 1000 random HiChIP deconvolution results in K562. Source data is available upon request.

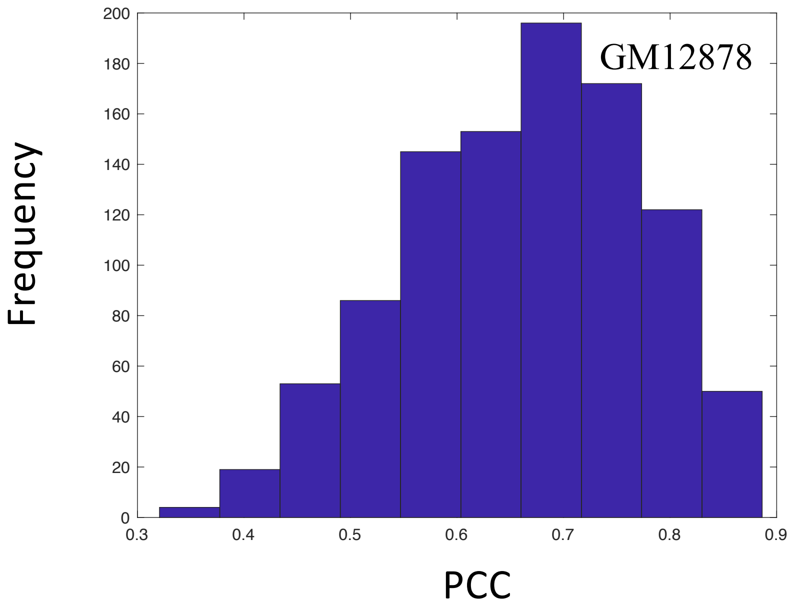

Supplementary Figure 6. The distribution of 1000 random RNA-seq deconvolution results in GM12878. Source data is available upon request.

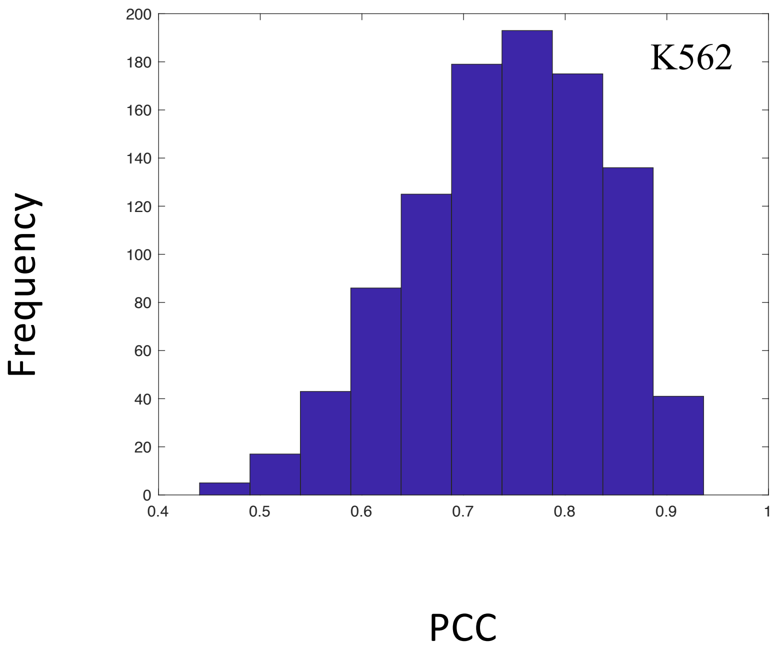

Supplementary Figure 7. The distribution of 1000 random RNA-seq deconvolution results in K562. Source data is available upon request.

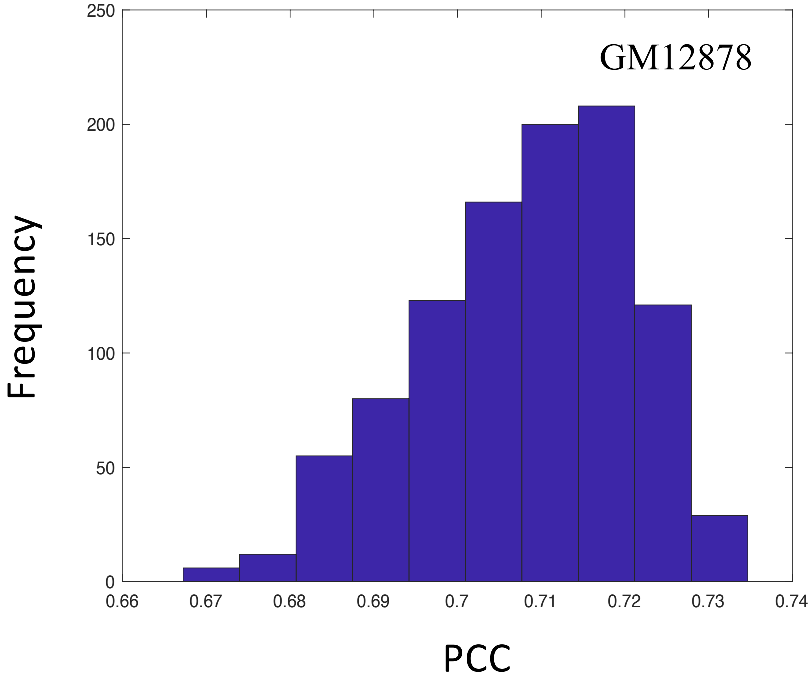

Supplementary Figure 8. The distribution of 1000 random ATAC-seq deconvolution results in GM12878. Source data is available upon request.

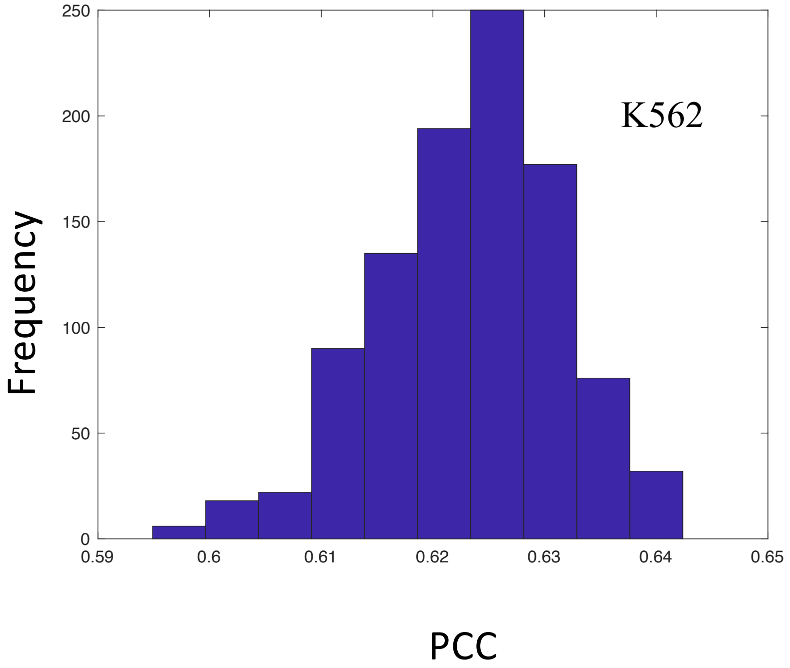

Supplementary Figure 9. The distribution of 1000 random ATAC-seq deconvolution results in K562. Source data is available upon request.

A

B

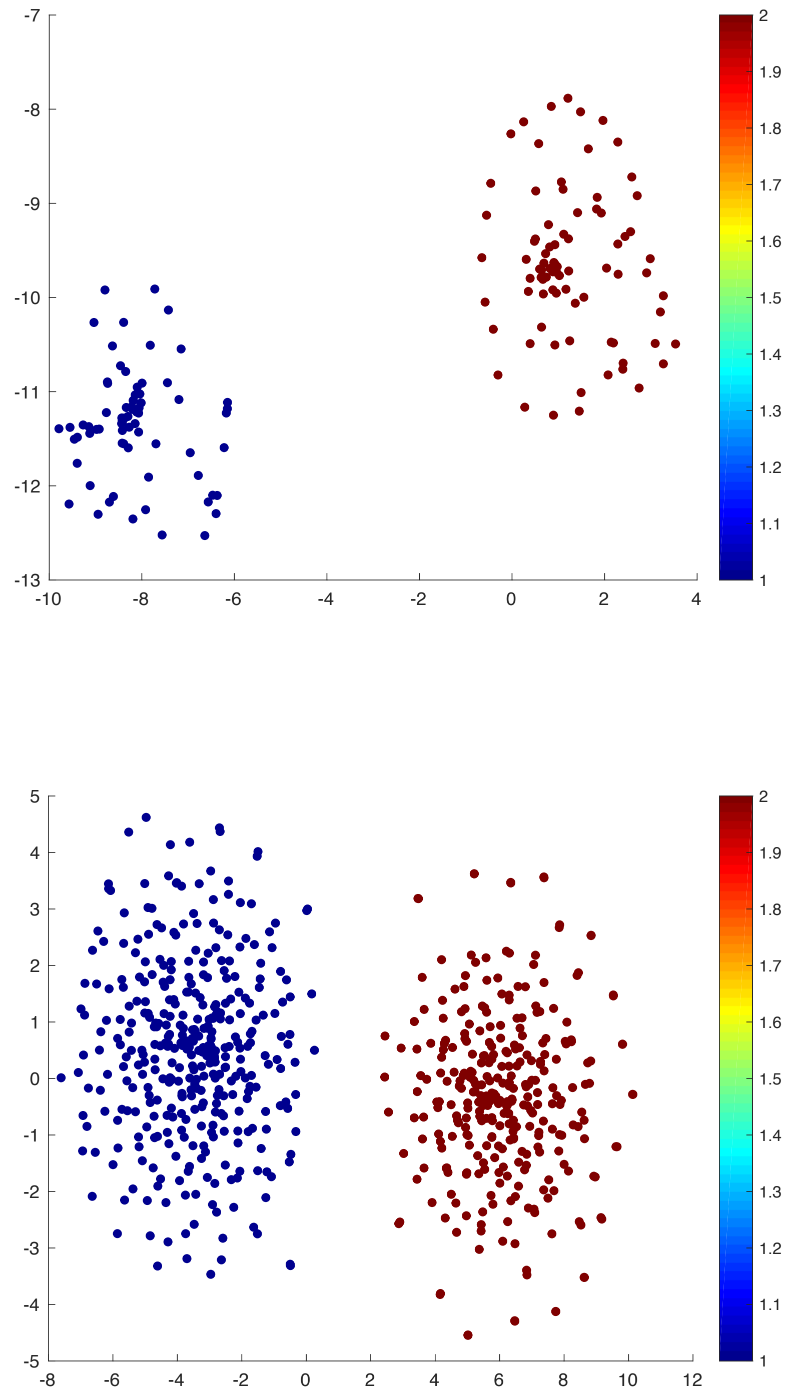

Supplementary Figure 10. The *t*-SNE plot for simulated GM12878 and K562 mixture for A) scRNA-seq, and B) scATAC-seq without dropout. The blue points are from GM12878 and the red points are from K562. Source data is available upon request.

A

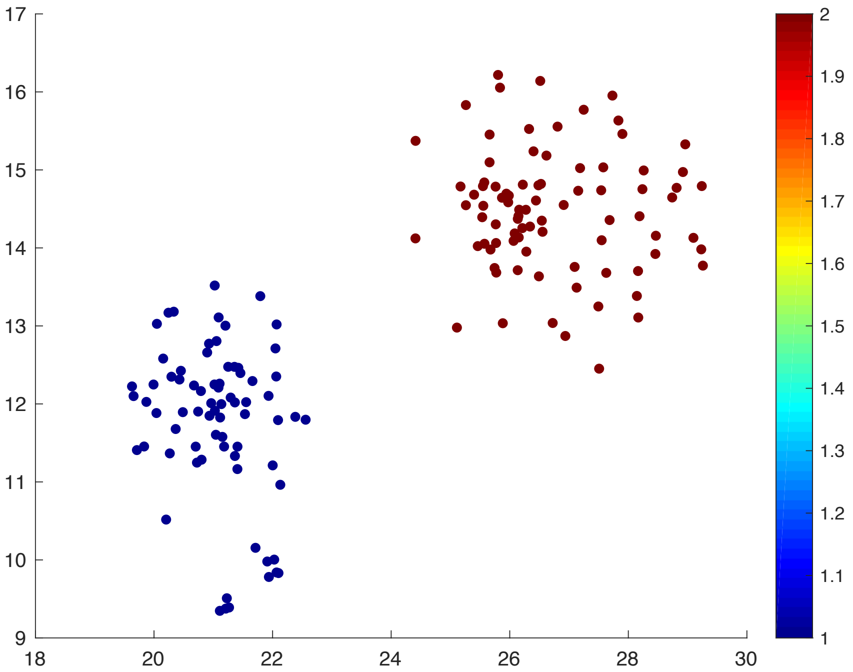

B

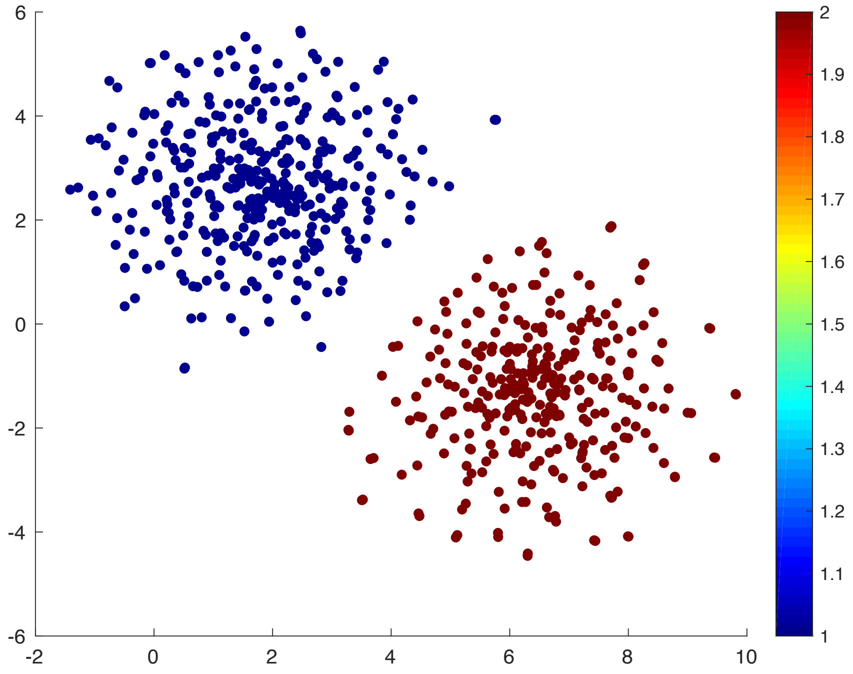

Supplementary Figure 11. The *t*-SNE plot for simulated GM12878 and K562 mixture for A) scRNA-seq, and B) scATAC-seq with 50% "site" level dropout. The blue points are from GM12878 and the red points are from K562. Source data is available upon request.

A

B

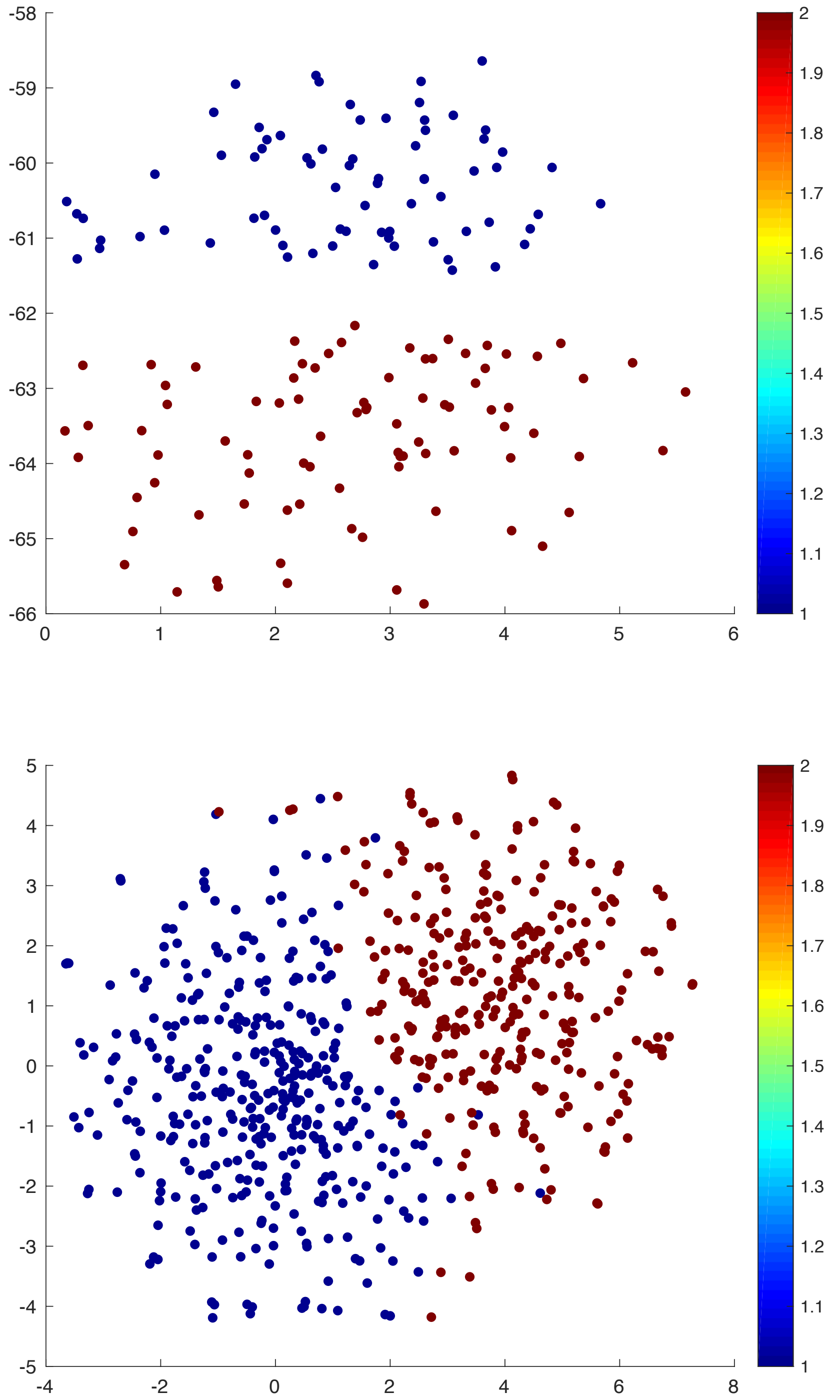

Supplementary Figure 12. The *t*-SNE plot for simulated GM12878 and K562 mixture for A) scRNA-seq, and B) scATAC-seq with 80% "site" level dropout. The blue points are from GM12878 and the red points are from K562. Source data is available upon request.

A

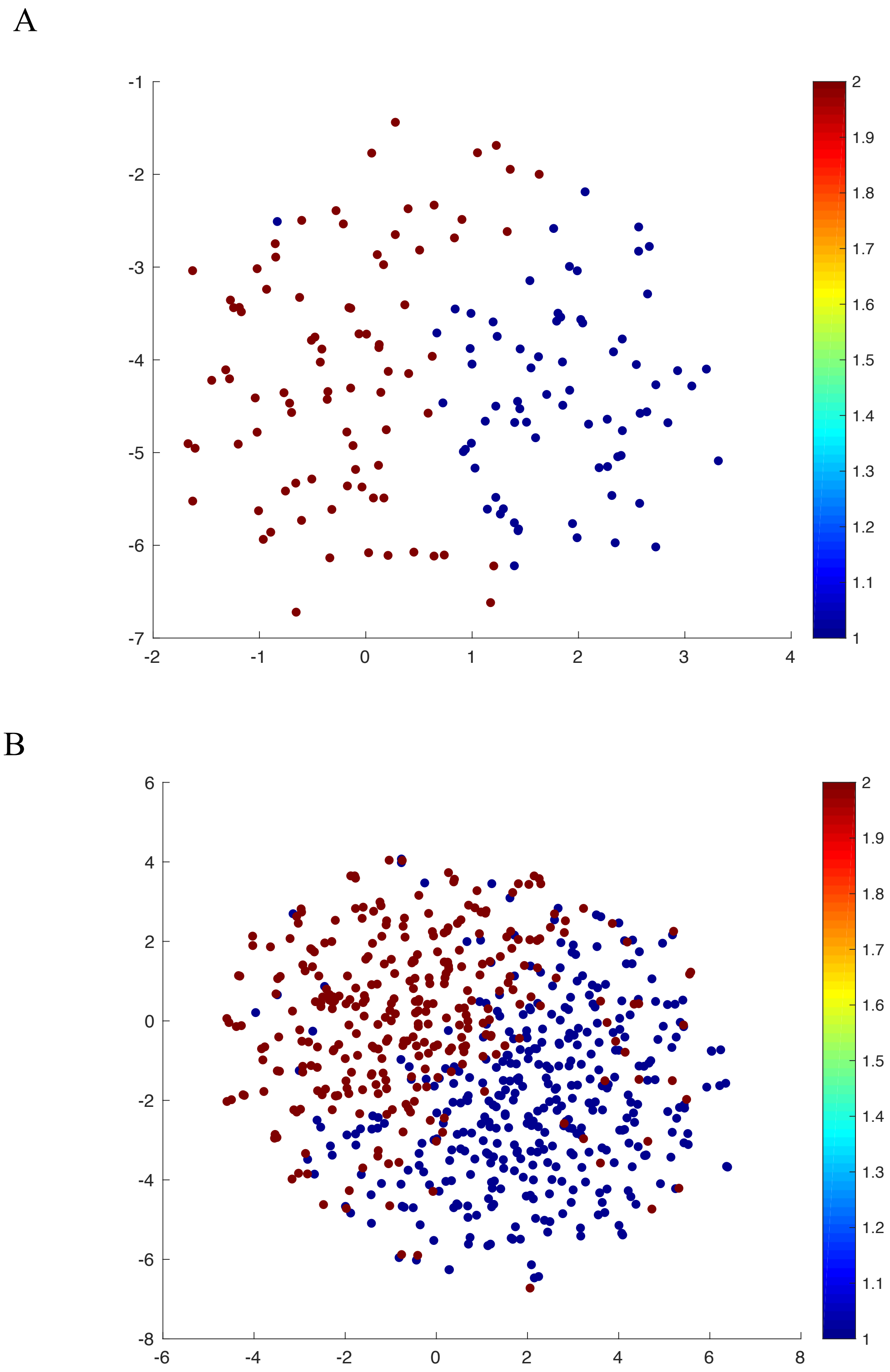

Supplementary Figure 13. The t-SNE plot for simulated GM12878 and K562 mixture for A) scRNA-seq, and B) scATAC-seq with 90% "site" level dropout. The blue points are from GM12878 and the red points are from K562. Source data is available upon request.

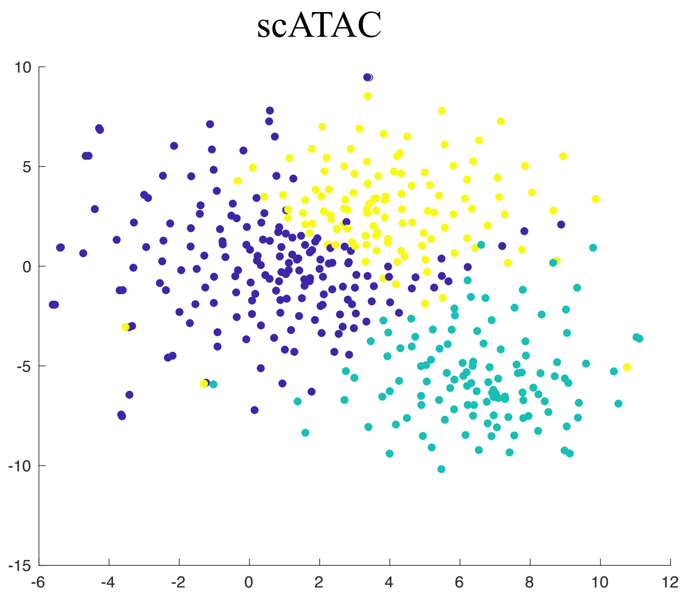

Supplementary Figure 14. The t-SNE plot for scATAC-seq clustering results in RA-day 4. Source data is available upon request.

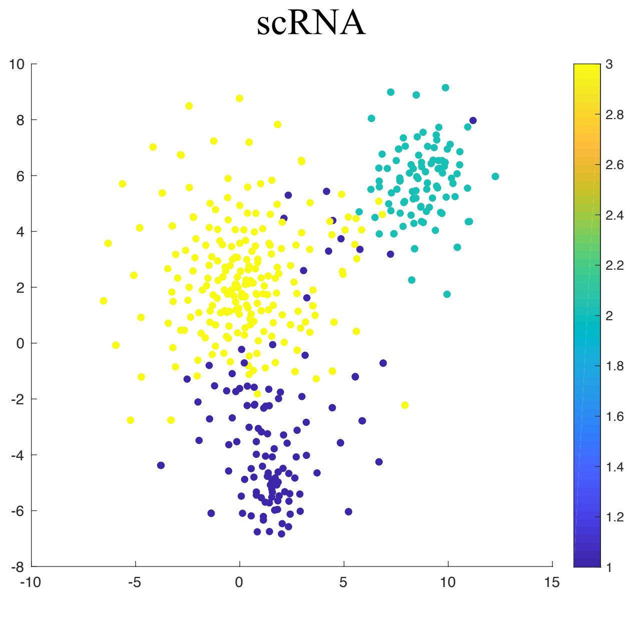

Supplementary Figure 15. The t-SNE plot for scRNA-seq clustering results in RA-day 4. Source data is available upon request.

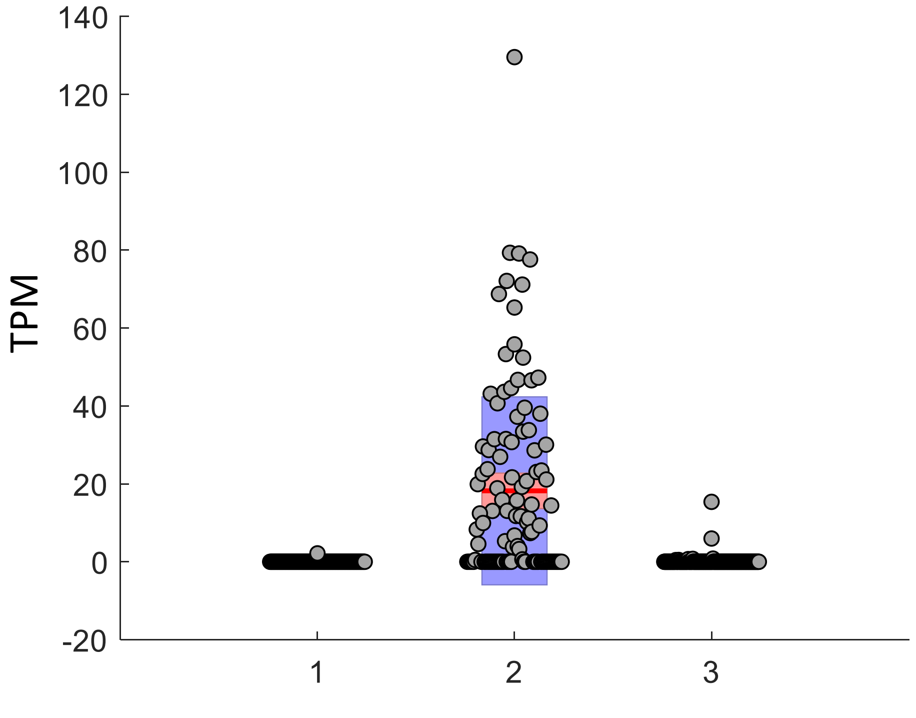

Supplementary Figure 16. CD38 expression level (TPM) in each cluster. We can see that CD38 mostly expressed in subpopulation 2 cells. Source data is available upon request.

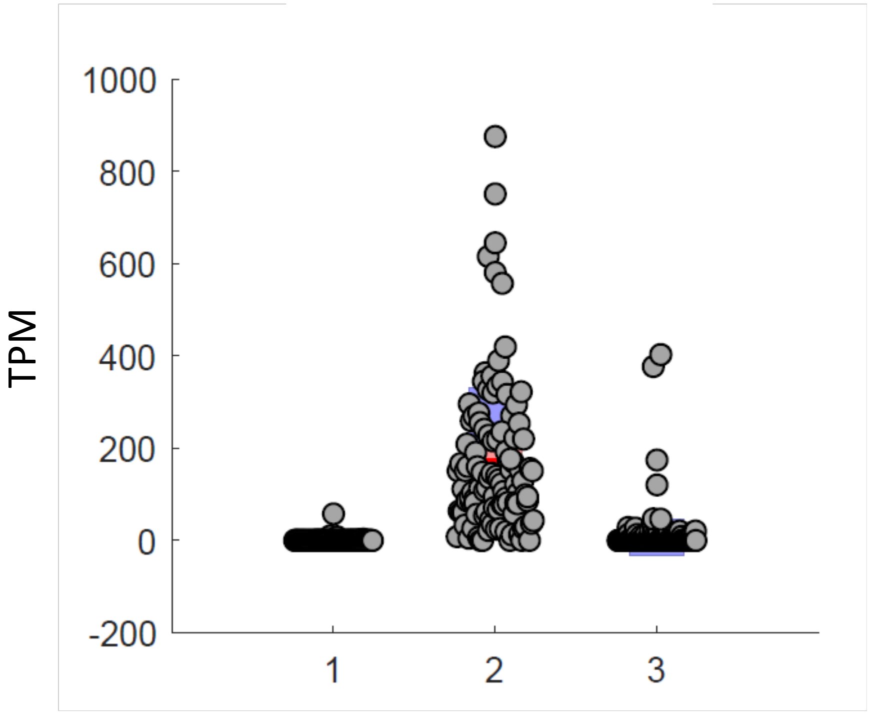

Supplementary Figure 17. EpCAM expression level (TPM) in each cluster. We can see that EpCAM mostly expressed in subpopulation 2 cells. Source data is available upon request.

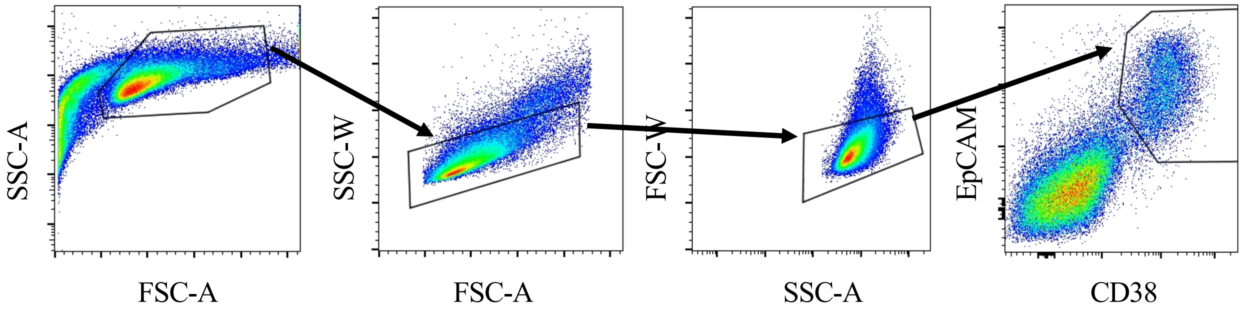

Supplementary Figure 18. Gating strategy to sort CD38 and EpCAM double positive cells from RA-day 4 sample for HiChIP experiment presented on Figure 2c.

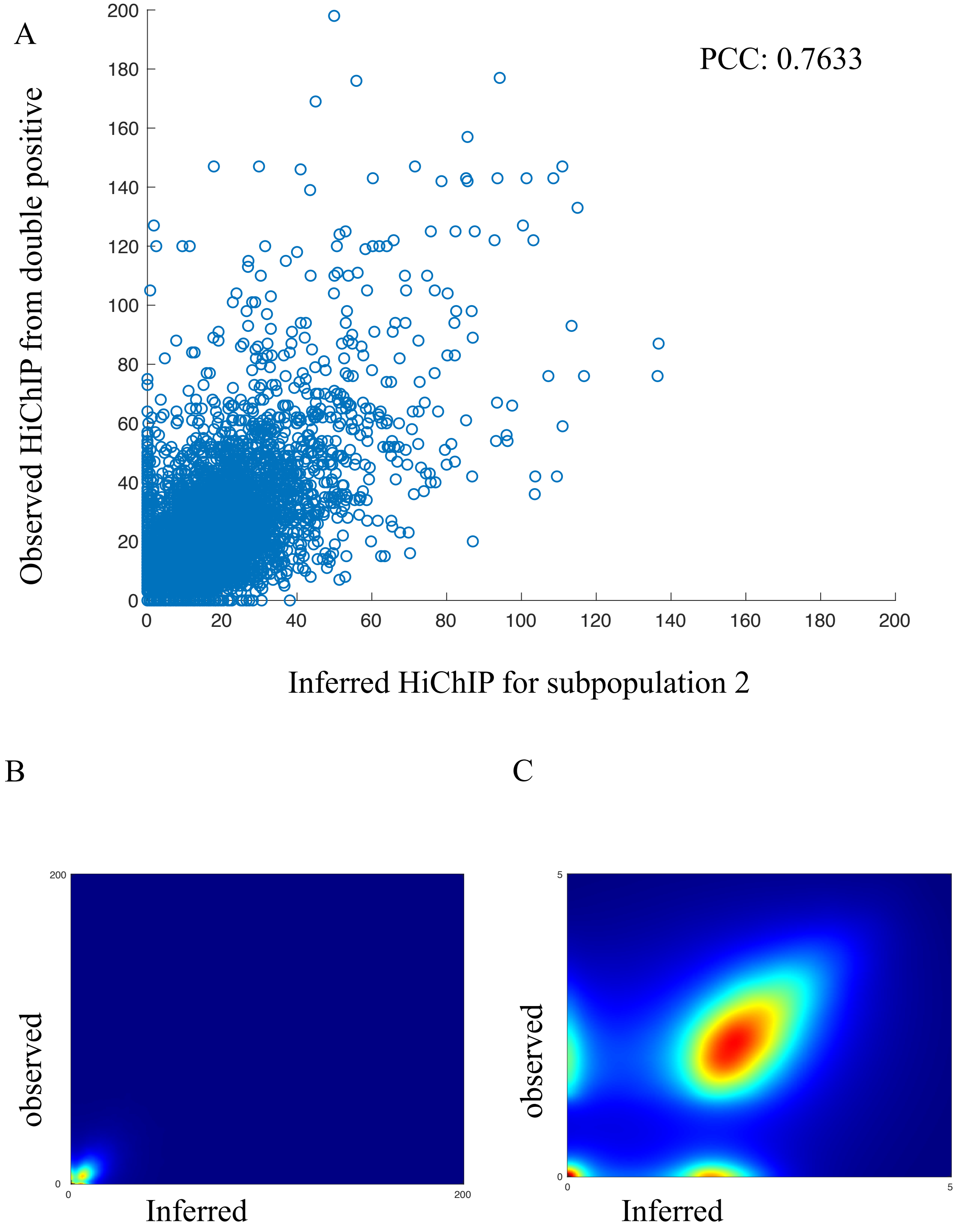

Supplementary Figure 19. A) Scatter plot between the HiChIP profile from EpCAM/CD38 double positive cells and the inferred HiChIP profile for population 2. The PCC is 0.7633. B) The contour plot of the original scatter plot. C) The contour plot of the log2 scale of the original scatter plot. Source data is available upon request.

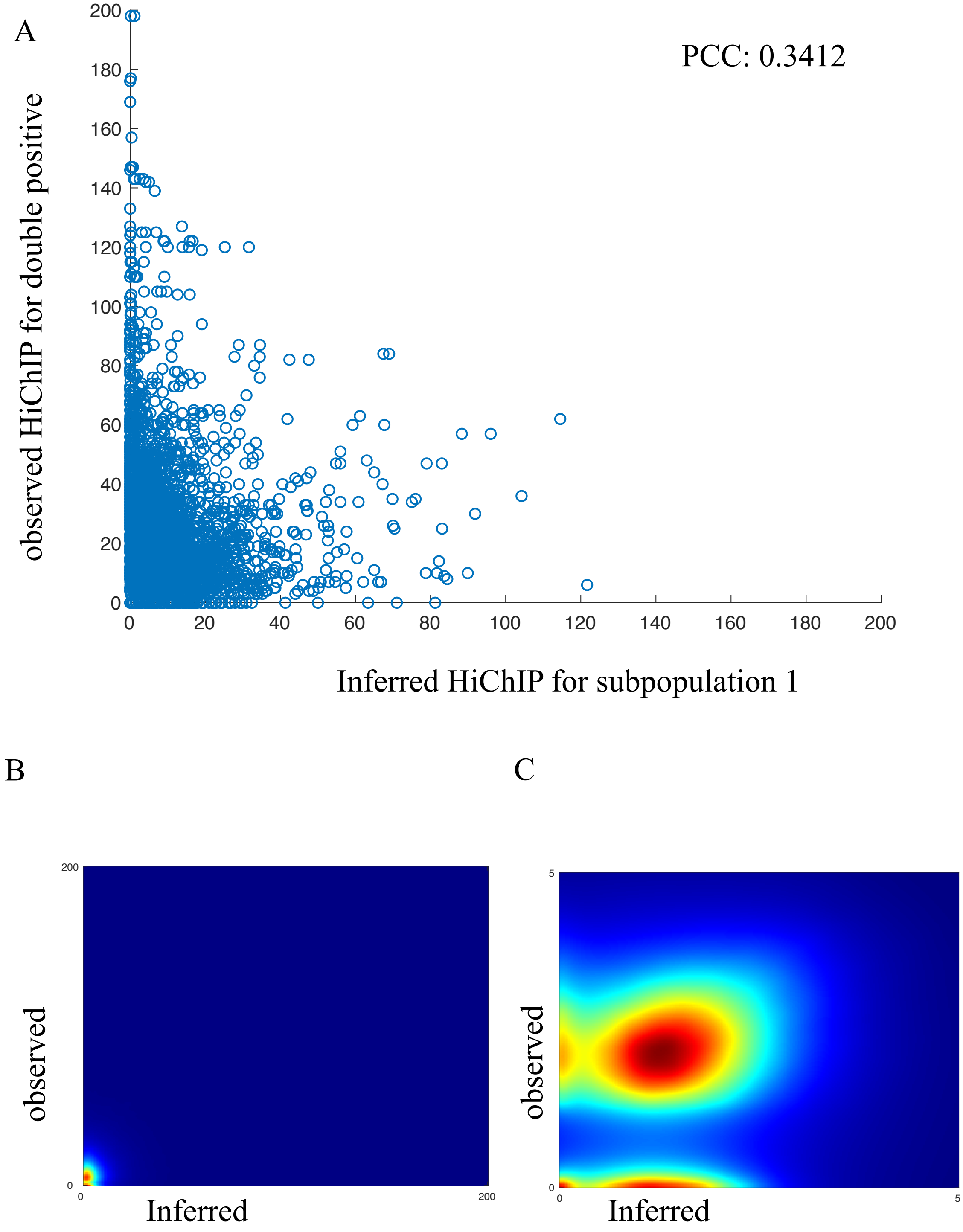

Supplementary Figure 20. A) Scatter plot between HiChIP profiles from EpCAM/CD38 double positive cells and the inferred HiChIP profile for subpopulation 1. The PCC is 0.3412. B) The contour plot of the original scatter plot. C) The contour plot of the log2 scale of the original scatter plot. Source data is available upon request.

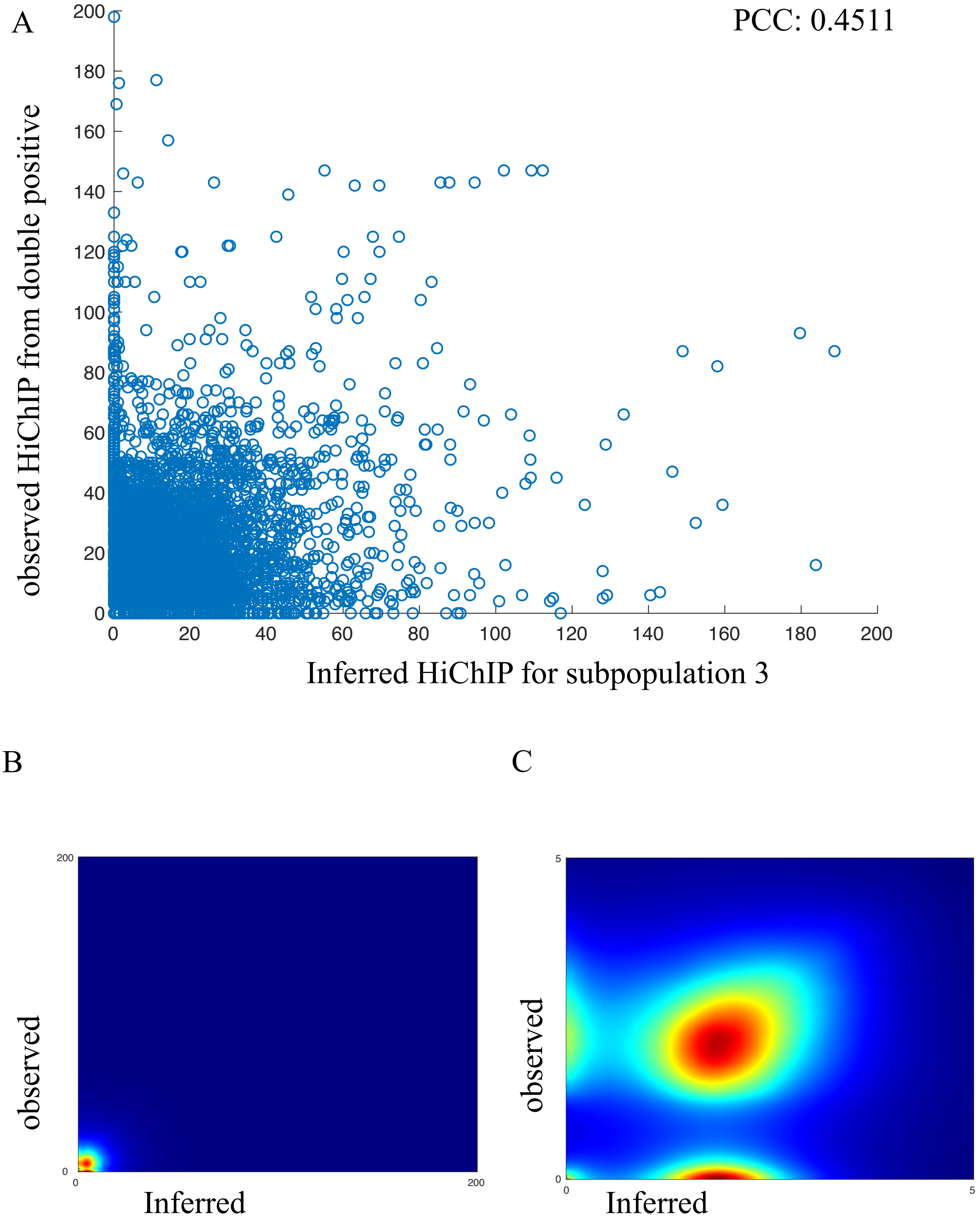

Supplementary Figure 21. A) Scatter plot between HiChIP profiles from observed cluster 2 and predicted cluster 3. The PCC is 0.4511. B) The contour plot of the original scatter plot. C) The contour plot of the log2 scale of the original scatter plot. Source data is available upon request.

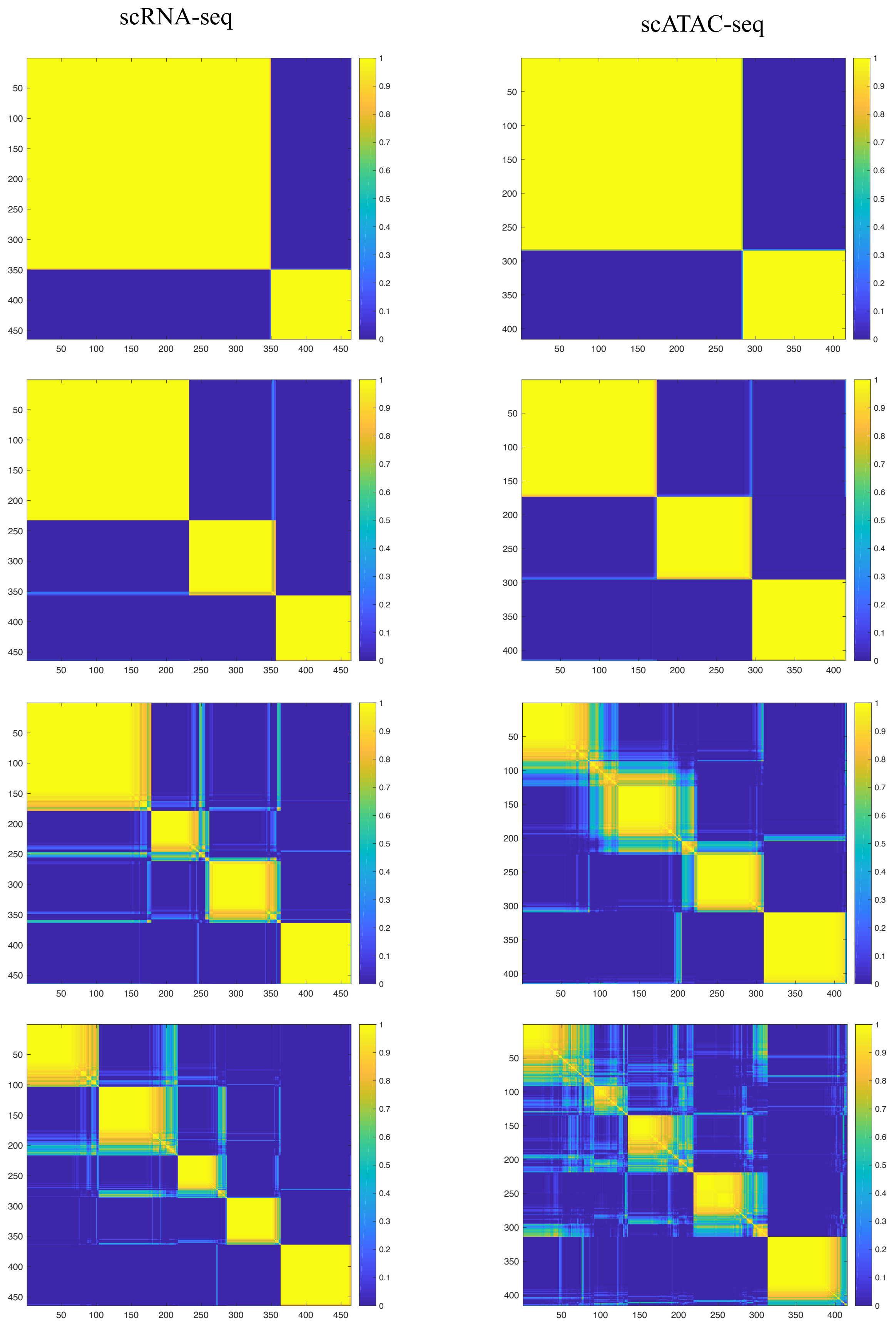

Supplementary Figure 22. Clustering stability of NF clustering in RA day 4 single cell data for k=2 to 5. Samples are hierarchically clustered by using distances derived from consensus clustering matrix entries, colored from 0 (deep blue, samples are never in the same cluster) to 1 (dark yellow, samples are always in the same cluster). Source data is available upon request.

Supplementary Table 1. The deconvolution performance using W matrix and using mean singlecell profile (input: scRNA-seq, scATAC-seq and scHi-C).

cell type	data type	W matrix	mean singlecell profile
K562	scRNA-seq	0.84	0.98
	scATAC-seq	0.82	0.98
	scHi-C	0.87	0.92
GM12878	scRNA-seq	0.86	0.99
	scATAC-seq	0.81	0.99
	scHi-C	0.89	0.95

Supplementary Table 2. The comparison results for the two methods for the estimation of subpopulation profiles for bulk data type in terms of PCC and time complexity.

Methods	Dropout rates	PCC for GM12878	PCC for K562	time
Simple method	0	0.9462	0.8562	~ 3 mins
	50%	0.9201	0.8502	~ 3 mins
	80%	0.8923	0.8157	~ 3 mins
	90%	0.8874	0.7701	~ 3 mins
Poisson-based method	0	0.9503	0.8603	~ 24 hours
	50%	0.9277	0.8547	~ 24 hours
	80%	0.8994	0.8160	~ 24 hours
	90%	0.8860	0.7821	~ 24 hours

Supplementary Table 3. The deconvolution performance of DC3 with different input combinations under 50% dropout rate.

Input combinations	HiChIP		RNA-seq		ATAC-seq	
	K562	GM12878	K562	GM12878	K562	GM12878
scRNA-seq, scATAC-seq and scHi-C	0.88 ± 0.01	0.91 ± 0.01	0.97 ± 0.02	0.93 ± 0.02	0.95 ± 0.03	0.97 ± 0.01
scRNA-seq, scATAC-seq and bulk Hi-C	0.85 ± 0.00	0.92 ± 0.00	0.96 ± 0.02	0.93 ± 0.01	0.95 ± 0.05	0.97 ± 0.02
scRNA-seq, bulk ATAC-seq, bulk Hi-C	0.75 ± 0.07	0.84 ± 0.08	0.90 ± 0.02	0.91 ± 0.07	0.82 ± 0.05	0.82 ± 0.05
bulk RNA-seq, scATAC-seq, bulk Hi-C	0.70 ± 0.08	0.80 ± 0.05	0.82 ± 0.05	0.84 ± 0.06	0.85 ± 0.01	0.84 ± 0.08
random deconvolution	0.61 ± 0.12	0.76 ± 0.10	0.76 ± 0.11	0.74 ± 0.08	0.62 ± 0.12	0.71 ± 0.08

Supplementary Table 4. The deconvolution performance of DC3 with different input combinations under 80% dropout rate.

Input combinations	HiChIP		RNA-seq		ATAC-seq	
	K562	GM12878	K562	GM12878	K562	GM12878
scRNA-seq, scATAC-seq and scHi-C	0.86 ± 0.02	0.89 ± 0.01	0.93 ± 0.04	0.91 ± 0.01	0.90 ± 0.05	0.89 ± 0.07
scRNA-seq, scATAC-seq and bulk Hi-C	0.82 ± 0.00	0.90 ± 0.00	0.91 ± 0.05	0.90 ± 0.02	0.90 ± 0.05	0.89 ± 0.05
scRNA-seq, bulk ATAC-seq, bulk Hi-C	0.70 ± 0.10	0.84 ± 0.09	0.82 ± 0.08	0.86 ± 0.09	0.79 ± 0.09	0.79 ± 0.09
bulk RNA-seq, scATAC-seq, bulk Hi-C	0.63 ± 0.11	0.74 ± 0.10	0.79 ± 0.09	0.84 ± 0.10	0.78 ± 0.11	0.79 ± 0.08
random deconvolution	0.61 ± 0.12	0.76 ± 0.10	0.76 ± 0.11	0.74 ± 0.08	0.62 ± 0.12	0.71 ± 0.08

Supplementary Table 5. The deconvolution performance of DC3 with different input combinations under 90% dropout rate.

Input combinations	HiChIP		RNA-seq		ATAC-seq	
	K562	GM12878	K562	GM12878	K562	GM12878
scRNA-seq, scATAC-seq and scHi-C	0.80 ± 0.03	0.78 ± 0.02	0.90 ± 0.07	0.86 ± 0.01	0.80 ± 0.14	0.80 ± 0.10
scRNA-seq, scATAC-seq and bulk Hi-C	0.77 ± 0.01	0.89 ± 0.01	0.89 ± 0.06	0.85 ± 0.02	0.79 ± 0.10	0.81 ± 0.08
scRNA-seq, bulk ATAC-seq, bulk Hi-C	0.67 ± 0.11	0.77 ± 0.10	0.76 ± 0.15	0.79 ± 0.05	0.70 ± 0.11	0.70 ± 0.11
bulk RNA-seq, scATAC-seq, bulk Hi-C	0.59 ± 0.09	0.65 ± 0.11	0.70 ± 0.11	0.80 ± 0.09	0.69 ± 0.11	0.68 ± 0.15
random deconvolution	0.61 ± 0.12	0.76 ± 0.10	0.76 ± 0.11	0.74 ± 0.08	0.62 ± 0.12	0.71 ± 0.08

Supplementary Table 6. The clustering results of DC3.

Methods	Dropout rates	Error rates	sd
DC3	0	0	0
DC3	50%	0	0
DC3	80%	0.00354191	0.00135301
DC3	90%	0.0377804	0.0126026
coupleNMF	0	0	0
coupleNMF	50%	0.00354191	0.00025171
coupleNMF	80%	0.01416765	0.00368583
coupleNMF	90%	0.17119244	0.04732597
NMF	0	0	0
NMF	50%	0.00590319	0.00084345
NMF	80%	0.02361275	0.00635407
NMF	90%	0.3364817	0.10871913

Supplmentary Table 7. The mean performance of DC3 under different dropout rate (50 runs). Same scRNA-seq, same scATAC-seq, random HiChIP.

Dropout rate	0%	50%	80%	90%
joint clustering performance (error rate)	0.001/892	160.44/892	170.60/892	296.98/892

Supplementary Table 8. The mean performance of DC3 under different dropout rate (50 runs). Same scRNA-seq, random scATAC-seq, same HiChIP.

Dropout rate	0%	50%	80%	90%
clustering performance for scRNA-seq (error rate)	0.02/146	0.20/146	5.34/146	9.55/146
deconvolution performance for GM1278 (PCC)	0.8	0.79	0.77	0.77
deconvolution performance for K562 (PCC)	0.71	0.7	0.66	0.65

Supplementary Table 9. The mean performance of DC3 under different dropout rate (50 runs). random scRNA-seq, same scATAC-seq, same HiChIP.

Dropout rate	0%	50%	80%	90%
clustering performance for scATAC-seq (error rate)	1.52/746	16.22/746	248.81/746	388.85/746
deconvolution performance for GM1278 (PCC)	0.74	0.71	0.68	0.68
deconvolution performance for K562 (PCC)	0.63	0.63	0.59	0.5

Supplementary Table 10. The key regulators for subpopulations 1.

TFName	Expression	Expression Fold Change	Expression p-value	Motif enrichment p-value	Motif enrichmet Fold Change	Score
Isl1	31.85442392	17.17653323	7.97E-05	6.69E-185	1.54	1457.372
Lhx1	32.21523274	11.48052139	9.32E-21	1.06E-275	2.0506	1377.727
Hoxb5	135.7075345	8.503216324	2.43E-29	1.17E-299	1.6416	1336.449
Hoxc6	37.72494719	9.940104007	2.43E-16	1.17E-299	1.6416	1161.579
Hoxb2	113.190633	7.13553279	3.21E-29	1.17E-299	1.6416	1080.448
Hoxb6	111.6656869	6.804253507	2.27E-24	1.17E-299	1.6416	1027.363
Lhx3	27.40002464	8.527398234	9.05E-08	1.17E-299	1.8984	980.7257
Bhlhe23	22.53960016	16.00475421	2.06E-14	5.09E-130	1.3924	978.5886
Neurod1	64.02820785	6.976489563	1.91E-24	6.82E-196	1.7932	786.0793
Pbx3	65.79698358	8.154033016	2.14E-15	4.98E-129	1.7318	736.7748
Hoxa2	105.0108506	4.742241672	9.09E-19	1.17E-299	1.6416	706.7955
Hoxc4	38.0945218	5.968808716	2.12E-23	1.17E-299	1.6416	699.3141
Sox11	116.2905543	9.747193107	4.59E-43	2.95E-74	1.3677	671.9143
Hoxb7	63.76910125	5.023166076	4.17E-13	1.17E-299	1.6416	669.5672
Hoxb9	50.65988514	5.193252072	3.58E-14	7.16E-203	2.4677	660.0905
Hoxd3	22.59913441	6.431555347	1.17E-18	1.17E-299	1.6416	649.776
Hoxa5	147.7229386	3.838735975	8.59E-14	1.17E-299	1.6416	613.6682
Onecut2	13.23310669	8.729281322	5.26E-34	4.44E-125	2.0258	530.8064
Pbx1	27.78799328	6.348047243	1.47E-33	4.98E-129	1.7318	458.6862
Hoxa4	63.83732817	3.125786222	1.45E-10	1.17E-299	1.6416	416.7595
Hoxc5	32.40975552	3.707660077	1.72E-10	1.17E-299	1.6416	415.7754
Meis2	36.5109572	6.683012906	1.72E-23	2.68E-65	1.9133	388.4404
Neurog1	53.6786764	7.432754527	2.66E-12	2.38E-58	1.3984	385.1765
Ascl1	11.05696903	7.961432491	1.70E-10	4.36E-121	1.37	367.202
Sox4	47.56050913	6.289391969	1.51E-31	2.95E-74	1.3677	353.3115
Hoxb4	10.71520523	4.373633597	7.80E-25	1.17E-299	1.6416	343.9762
Hoxa3	15.09030742	3.544901005	3.20E-10	1.17E-299	1.6416	314.7492
Hoxa7	18.39413096	3.272203908	4.86E-05	1.17E-299	1.6416	310.0666
Ebf1	13.12398404	7.219813196	7.99E-31	3.49E-88	1.3676	301.6299
Nr2f6	54.69723643	5.726966775	2.64E-07	2.48E-60	1.246	286.233
Pax8	17.68477072	12.14984091	3.75E-11	1.79E-25	1.2241	282.4568
Klf7	11.91218968	5.372525616	2.69E-17	4.13E-114	1.6152	268.3317
Lhx5	41.53146684	10.60046575	2.69E-17	1.40E-12	1.8103	265.6695
Meis3	75.06413577	3.667588657	8.27E-09	2.68E-65	1.9133	254.7503
Pou3f2	27.34305268	4.086403545	3.30E-15	3.84E-111	1.4588	250.2337
Neurog2	17.87158573	3.098483922	2.25E-05	6.82E-196	1.7932	245.6642
Tcf12	43.48743266	3.486479768	1.45E-05	3.71E-106	1.4538	236.3371
Nr2f1	42.14549591	4.551928849	4.35E-21	2.48E-60	1.246	213.0533
Tcf3	19.7135422	3.652176263	5.99E-08	3.71E-106	1.4538	197.7045
Pax6	24.44756273	5.623667876	1.20E-13	1.79E-25	2	184.7445
Mxi1	12.78081767	1.67332067	1.21E-07	5.62E-148	4.4627	162.3399
Nhlh1	11.03184761	4.815181213	2.03E-17	2.38E-58	1.4603	158.5189
Id2	191.9704098	4.034888555	1.63E-12	1.52E-17	1.3183	144.2446
Nfil3	22.79442713	3.94957077	7.06E-08	2.06E-36	1.6052	136.6862
Tcf4	11.11416424	3.012419388	1.05E-08	3.71E-106	1.4538	134.2105
Sox2	18.36882905	2.883597386	1.13E-04	3.16E-82	1.3808	130.7924
Zbtb18	22.46571171	3.595500175	3.25E-05	2.10E-38	1.4487	120.9309
Meis1	24.70426598	2.214668882	1.72E-06	2.68E-65	1.9133	115.3011
Nr2f2	12.44229653	3.383193449	6.73E-09	2.48E-60	1.246	109.2975
Jund	53.85528529	1.905310789	1.13E-08	2.15E-41	2.0559	100.6541
Tcf15	41.27140253	6.573272042	4.72E-05	1.31E-07	1.1148	98.34121
Sox9	11.63714326	2.62741712	4.86E-10	2.95E-74	1.3677	96.42527
Rara	23.05251752	2.713560451	2.66E-07	1.91E-30	1.7989	91.03106
Insm1	14.66310086	7.783968936	4.93E-14	1.25E-05	1.17	74.01002
Zeb1	11.00184038	5.049714812	7.51E-20	1.44E-14	1.0802	70.00757
Tead2	20.03917477	2.167203145	4.58E-04	2.27E-48	1.0989	68.91926
Gtf2i	21.52089593	1.943078191	2.25E-07	1.46E-15	1.1201	35.59119
Patz1	28.13167533	3.472098833	4.23E-10	1.23E-04	1.0994	35.02668

Supplementary Table 11. The key regulators for subpopulations 2.

TFName	Expression	Expression Fold Change	Expression p-value	Motif enrichment p-value	Motif enrichmet Fold Change	Score
Gata4	51.62684755	46.15681244	4.21E-107	1.76E-299	3.0541	7971.847338
Sox17	67.60876802	53.40043411	2.15E-105	1.76E-299	1.6833	7305.271451
Foxa2	56.5700441	46.83487758	3.76E-74	1.76E-299	1.999	6692.452527
Elf3	45.24846766	84.69655642	2.75E-59	3.56E-73	1.6458	5115.613519
Hnf1b	20.69603134	28.75019669	7.00E-41	1.02E-212	6.2308	4638.667878
Gata6	29.09774817	25.49988696	2.16E-63	1.76E-299	3.0541	3783.202109
Atf3	150.2371675	23.18363752	2.62E-40	7.55E-180	1.9623	3147.151224
Klf5	27.3357004	26.86994132	4.35E-72	1.43E-289	1.5454	2738.895479
Sox7	31.67304129	23.14706544	7.61E-29	1.76E-299	1.6833	2610.990511
Isx	28.92232062	52.81972415	7.07E-32	3.25E-70	1.4016	2555.869278
Klf4	21.55475825	25.17495938	2.05E-68	1.43E-289	1.5454	2391.031352
Klf6	103.9438168	9.555359518	8.43E-42	1.43E-289	1.5454	1355.333974
Fos	104.7651902	6.752800964	2.41E-08	9.00E-201	2.1474	941.1944805
Pitx1	15.93368319	21.46867344	9.98E-23	3.25E-70	1.4016	864.8221194
Nfe2l2	77.37700837	6.574314264	6.98E-24	5.16E-105	3.8824	832.395209
Jun	34.64430693	6.387583101	2.32E-34	9.49E-206	2.2929	714.0185519
Junb	29.42674871	6.129797221	2.95E-23	9.49E-206	2.2929	654.8563672
Zfp42	29.15398999	26.4514452	1.64E-40	1.72E-22	1.1022	636.6817925
Creb3	246.7312027	7.293102748	2.52E-36	2.17E-33	2.2951	502.1807704
Tcf7l2	30.40657136	4.454083406	8.20E-22	7.09E-173	2.1912	430.1995642
Msx2	19.76117154	15.02876631	1.56E-23	2.26E-35	1.2184	427.2740597
Creb3l1	33.48003282	17.06559622	2.09E-36	1.57E-15	1.3333	387.2440095
Xbp1	257.7422433	5.195473191	2.32E-29	2.09E-31	2.1912	341.4424258
Bhlhe40	49.67815137	7.806047791	4.93E-29	1.87E-27	1.521	281.8739294
Elf1	17.20990804	5.56096756	9.31E-27	4.33E-88	1.5092	267.3343758
Pitx2	10.62115564	6.681221746	2.43E-16	3.25E-70	1.4016	233.3268367
Creb3l2	11.8606878	13.99470169	2.66E-57	1.57E-15	1.3333	229.101348
Jund	45.00922717	1.592351906	7.18E-06	1.21E-233	2.777	223.7018887
Hes1	100.1546504	8.472937963	3.14E-30	1.49E-10	1.1546	190.0880582
Atf1	16.51965151	3.251551155	1.13E-09	5.32E-107	1.6485	177.7845326
Egr1	18.84298041	3.060581175	1.00E-07	2.74E-45	1.3091	100.7640357
Mlx	53.2332989	2.27188497	2.05E-06	2.04E-30	1.8699	97.52325543
Hoxa1	29.3321636	1.442634589	1.58E-04	3.77E-87	1.3166	75.75451045
Rela	21.3622289	2.652781039	7.06E-10	1.77E-23	1.2101	62.3998679
E2f4	65.39594062	1.409091009	2.76E-05	1.82E-26	1.2769	48.89750491
Irf9	10.01080422	2.986464059	1.22E-11	1.42E-08	1.5	35.46472561
Nr1h2	26.22265568	1.538234417	5.52E-05	1.61E-17	1.1538	32.27468878
Irf3	20.51869817	2.359724001	1.64E-07	1.37E-07	1.2105	30.11519749
Smad2	62.93923311	1.68066331	6.91E-05	1.42E-08	1.086	29.43464737
Bbx	13.94321532	3.030603034	1.11E-17	1.24E-04	1.2123	25.73561605
Tgif2	14.36986273	1.821105061	2.19E-05	1.51E-12	1.0726	25.5614888
Zfx	11.71761242	1.763256711	1.45E-07	0.000123592	1.078	13.27766322

Supplementary Table 12. The key regulators for subpopulations 3.

TFName	Expression	Expression Fold Change	Expression p-value	Motif enrichment p-value	Motif enrichmet Fold Change	Score
Rfx4	24.51136087	5.89313904	3.93E-24	3.05E-299	4.2208	977.5287398
Sox2	44.39428899	6.969157116	1.17E-51	3.05E-299	2.0748	954.6953213
Hoxa2	118.5276174	5.35265264	1.99E-38	1.43E-266	1.5672	753.9962988
Sox3	14.75253643	6.607268986	2.48E-38	3.05E-299	2.0748	654.0410329
Pou3f2	33.43497082	4.996837218	1.10E-39	6.44E-191	2.7057	578.7541542
Hoxa5	149.0985926	3.874483791	3.29E-36	1.43E-266	1.5672	571.7598505
Hoxb2	65.35331944	4.119870535	1.48E-45	1.43E-266	1.5672	508.9380923
Sox11	45.84438056	3.84256514	2.46E-43	3.05E-299	1.8675	503.5151703
Pax6	41.24518988	9.48762263	2.41E-58	2.52E-43	2.1944	495.4132723
Pou3f4	19.02314324	4.814638087	1.43E-23	6.44E-191	2.7057	472.2192789
Hoxb6	62.81974186	3.827867458	6.79E-39	1.43E-266	1.5672	468.4778223
Hoxb7	49.9386659	3.933726641	6.90E-17	1.43E-266	1.5672	455.3187713
Sox9	18.62848944	4.205912996	2.03E-31	3.05E-299	1.8675	426.5055717
Sox21	19.19429348	3.929798665	3.68E-25	3.05E-299	2.0748	424.0515101
Hoxb5	55.24133596	3.461333459	4.81E-39	1.43E-266	1.5672	410.7343735
Hoxb9	39.26648376	4.025290376	8.82E-21	5.57E-169	2.167	409.7881961
Hoxd3	16.64626877	4.737411488	1.15E-21	1.43E-266	1.5672	400.4537937
Hoxa4	53.37913525	2.613702206	1.71E-20	1.43E-266	1.5672	307.5597757
Hoxa7	19.36109009	3.44422005	1.37E-08	1.43E-266	1.5672	305.654133
Msx3	14.99897947	3.929131222	4.71E-05	7.98E-226	1.5846	296.8194238
Sox4	20.90299946	2.764208361	1.94E-21	3.05E-299	1.8675	290.631481
Hoxc4	19.27657188	3.02033376	5.65E-21	1.43E-266	1.5672	267.6667729
Hoxc6	12.99949328	3.425221899	1.30E-07	1.43E-266	1.5672	266.1838162
Hoxa3	13.42747229	3.15428034	1.91E-18	1.43E-266	1.5672	247.9252521
Pbx1	17.24918911	3.940502873	4.44E-35	4.59E-130	1.7342	247.2601328
Mycn	20.98719115	2.647214692	1.01E-10	6.16E-184	2.3465	244.7216424
Myc	10.66192111	2.694490056	2.90E-06	6.16E-184	2.3465	197.980851
Hoxc5	18.2987548	2.093368541	2.45E-07	1.43E-266	1.5672	182.4708203
Pbx3	20.56863226	2.549012088	1.34E-12	4.59E-130	1.7342	169.1505649
Nr2f1	42.20696193	4.558567493	2.03E-34	2.03E-30	1.5623	168.6869891
Dbx2	20.54529664	8.845728431	1.21E-15	1.46E-13	1.4244	167.5373526
Nfyb	38.75750469	1.955813728	7.30E-15	4.39E-106	2.2374	159.5457094
Tcf3	25.54605123	4.732720329	3.47E-31	2.26E-37	1.2515	151.6150314
Nr2f6	36.4309893	3.814435223	1.50E-13	2.03E-30	1.5623	135.7723095
Tead2	30.5934764	3.308633167	3.37E-27	2.75E-50	1.1046	121.95022
Tcf7l2	16.58245035	2.429067587	1.39E-19	4.25E-101	1.3349	116.2935649
Pax3	11.5672876	3.865187881	7.69E-08	2.16E-36	1.6773	109.1650926
Hey1	22.59665414	5.012322169	1.22E-11	1.71E-21	1.076	108.0564568
Hmga1	59.61007332	3.782969715	2.08E-42	1.63E-19	1.0705	100.4585996
Id2	178.5917133	3.753691316	7.40E-27	1.40E-10	1.2212	97.50940932
Neurog1	25.99316848	3.599210219	3.18E-11	1.81E-23	1.2259	90.35676761
Zfp410	65.87805891	2.102571597	2.07E-10	2.16E-36	1.2803	86.14928944
Meis2	17.53725883	3.210042576	3.18E-29	2.16E-36	1.113	85.1937793
Maz	12.8494547	2.085374493	2.15E-10	3.71E-83	1.2014	78.68852005
Rarb	61.2193008	1.936944758	3.25E-13	1.63E-19	2.4615	78.49498865
Fos	36.77059268	2.37010493	6.31E-06	1.98E-29	1.1198	70.39996893
E2f5	75.279447	2.51013215	1.24E-16	1.52E-16	1.2041	68.5053683
Nrf1	14.65048762	2.096342707	2.26E-07	2.36E-39	1.7314	68.02831748
Tcf12	26.12443292	2.094451231	1.76E-09	2.26E-37	1.2515	67.53784305
Rara	18.87512809	2.221831131	1.91E-09	1.63E-19	2.4615	65.16431165
Hmgn3	71.67091417	4.183388557	8.62E-31	1.26E-05	1.1127	60.38727496
Klf3	10.51211534	1.355649285	1.71E-05	4.01E-100	1.5764	59.81851866
Tgif2	16.15055619	2.046773874	4.35E-14	2.44E-41	1.1499	57.3503434
E2f4	88.72543672	1.91177333	3.24E-15	1.52E-16	1.2041	54.12952745
Meis3	32.20723188	1.573626035	8.36E-04	2.16E-36	1.113	50.10250349
Foxm1	11.54507019	4.505564047	1.51E-14	1.37E-08	1.0671	47.62632422
Zbtb18	21.39080992	3.423468699	8.27E-17	1.37E-08	1.1828	46.8256867
Tgif1	20.63049252	1.527207373	1.91E-06	2.44E-41	1.1499	46.28646107
Egr1	11.10661074	1.803997193	3.34E-06	2.29E-38	1.2702	44.87669917
Meis1	18.3376978	1.643923714	8.71E-08	2.16E-36	1.113	44.2610336
Id1	33.76329901	2.269614265	5.67E-07	1.40E-10	1.2212	40.305813
Id4	13.61925712	2.998243521	1.00E-07	1.40E-10	1.2212	40.24796091
E2f6	32.9667486	1.601995571	0.000105584	1.71E-21	1.1636	40.05309269
Gtf2i	23.73540401	2.143021649	4.01E-18	1.44E-12	1.1073	35.91545193
Usf1	52.77074207	1.447642231	2.18E-04	1.48E-14	1.2529	34.64137709
Plagl1	18.7931524	3.215450268	2.97E-10	1.26E-05	1.1818	33.31877199
Trim28	79.84517065	1.516881281	1.11E-06	1.37E-08	1.1777	29.25327324
Ccnt2	12.01071273	1.313725113	4.71E-04	1.93E-28	1.1283	27.1929179
Cbx5	37.99706094	2.286522798	3.68E-25	1.24E-04	1.2418	26.62247166
Hes1	20.60226046	1.742921313	4.12E-07	1.37E-08	1.0697	22.4095434
Patz1	18.75114363	2.314324446	4.01E-08	0.000123592	1.1029	20.67896416

Supplementary Table 13. Selecting tuning parameters μ_1 and μ_2. The values represent the sum connectivity of *K* subpopulation-specific subnetworks.

		μ_1				
		10^0	10^1	10^2	10^3	10^4
μ_2	10^0	1.7487	1.7444	1.8129	1.8001	1.8403
	10^1	1.8026	1.7474	1.8232	1.8295	1.8422
	10^2	1.8748	1.8451	1.8350	1.8433	1.8557
	10^3	1.8933	1.8800	1.9190	1.9317	1.8795
	10^4	1.9099	1.8996	1.9190	1.9321	1.9333
