# Supplementary material for: DC3 is a method for deconvolution and coupled clustering from bulk and single-cell genomics data
Source: Nat Commun. 2019 Oct 10;10:4613. doi: 10.1038/s41467-019-12547-1 (PMC6787340; doi:10.1038/s41467-019-12547-1)
Supplement: Supplementary file 3 — Reporting Summary [file 41467_2019_12547_MOESM3_ESM.pdf]

nature research

Corresponding author(s): Wing H. Wong

Last updated by author(s): 2019/08/26

Reporting Summary

Nature Research wishes to improve the reproducibility of the work that we publish. This form provides structure for consistency and transparency in reporting. For further information on Nature Research policies, see Authors & Referees and the Editorial Policy Checklist.

Statistics

For all statistical analyses, confirm that the following items are present in the figure legend, table legend, main text, or Methods section.

n/a	Confirmed	
☐	☒	The exact sample size (*n*) for each experimental group/condition, given as a discrete number and unit of measurement
☐	☒	A statement on whether measurements were taken from distinct samples or whether the same sample was measured repeatedly
☐	☒	The statistical test(s) used AND whether they are one- or two-sided<br>*Only common tests should be described solely by name; describe more complex techniques in the Methods section.*
☒	☐	A description of all covariates tested
☐	☒	A description of any assumptions or corrections, such as tests of normality and adjustment for multiple comparisons
☐	☒	A full description of the statistical parameters including central tendency (e.g. means) or other basic estimates (e.g. regression coefficient) AND variation (e.g. standard deviation) or associated estimates of uncertainty (e.g. confidence intervals)
☐	☒	For null hypothesis testing, the test statistic (e.g. *F*, *t*, *r*) with confidence intervals, effect sizes, degrees of freedom and *P* value noted<br>*Give P values as exact values whenever suitable.*
☒	☐	For Bayesian analysis, information on the choice of priors and Markov chain Monte Carlo settings
☒	☐	For hierarchical and complex designs, identification of the appropriate level for tests and full reporting of outcomes
☒	☐	Estimates of effect sizes (e.g. Cohen's *d*, Pearson's *r*), indicating how they were calculated

Our web collection on statistics for biologists contains articles on many of the points above.

Software and code

Policy information about availability of computer code

Data collection	MAC2 2.1.1.20160309, HiC-pro 2.10.0, hichipper 0.7.0,bowtie2-2.3.4.1-linux-x86_64, STAR/2.5.4b
Data analysis	We use DC3 to analyze the data https://github.com/SUwonglab/DC3.

For manuscripts utilizing custom algorithms or software that are central to the research but not yet described in published literature, software must be made available to editors/reviewers. We strongly encourage code deposition in a community repository (e.g. GitHub). See the Nature Research guidelines for submitting code & software for further information.

Data

Policy information about availability of data

All manuscripts must include a data availability statement. This statement should provide the following information, where applicable:

- Accession codes, unique identifiers, or web links for publicly available datasets
- A list of figures that have associated raw data
- A description of any restrictions on data availability

Figure 2, Figure 3, Figure4. The datasets generated during and analysed during the current study are available in (GEO GSE127807).

Field-specific reporting

Please select the one below that is the best fit for your research. If you are not sure, read the appropriate sections before making your selection.

☒ Life sciences ☐ Behavioural & social sciences ☐ Ecological, evolutionary & environmental sciences

For a reference copy of the document with all sections, see nature.com/documents/nr-reporting-summary-flat.pdf

Life sciences study design

All studies must disclose on these points even when the disclosure is negative.

Sample size	For RA-day4 HiChIP analysis, we did HiChIP on every one million cells and used total approximately 17 million cells. This number is enough for HiChIP based on the protocol developed by Mumbach et al. For validation, we use only 5 million cells since it is used for validate one subpopulation.
Data exclusions	We excluded 5 million cells for RA-day4 HiChIP that did not pass data quality filter.
Replication	There are total 12 replicates for bulk HiChIP data and there are total 5 replicates for cluster validation.
Randomization	No randomization for our experiment.
Blinding	No blinding is necessary for our experiment since we did not compare two different groups.

Reporting for specific materials, systems and methods

We require information from authors about some types of materials, experimental systems and methods used in many studies. Here, indicate whether each material, system or method listed is relevant to your study. If you are not sure if a list item applies to your research, read the appropriate section before selecting a response.

Materials & experimental systems

n/a	Involved in the study	
☐	☒	Antibodies
☐	☒	Eukaryotic cell lines
☒	☐	Palaeontology
☒	☐	Animals and other organisms
☒	☐	Human research participants
☒	☐	Clinical data

Methods

n/a	Involved in the study	
☒	☐	ChIP-seq
☐	☒	Flow cytometry
☒	☐	MRI-based neuroimaging

Antibodies

Antibodies used	EpCAM, CD38
Validation	*Describe the validation of each primary antibody for the species and application, noting any validation statements on the manufacturer's website, relevant citations, antibody profiles in online databases, or data provided in the manuscript.*

Eukaryotic cell lines

Policy information about cell lines

Cell line source(s)	*State the source of each cell line used.*
Authentication	*Describe the authentication procedures for each cell line used OR declare that none of the cell lines used were authenticated.*
Mycoplasma contamination	*Confirm that all cell lines tested negative for mycoplasma contamination OR describe the results of the testing for mycoplasma contamination OR declare that the cell lines were not tested for mycoplasma contamination.*
Commonly misidentified lines (See ICLAC register)	*Name any commonly misidentified cell lines used in the study and provide a rationale for their use.*

Flow Cytometry

Plots

Confirm that:

☒ The axis labels state the marker and fluorochrome used (e.g. CD4-FITC).

☒ The axis scales are clearly visible. Include numbers along axes only for bottom left plot of group (a 'group' is an analysis of identical markers).

☒ All plots are contour plots with outliers or pseudocolor plots.

☒ A numerical value for number of cells or percentage (with statistics) is provided.

Methodology

Sample preparation	Mouse embryonic stem cells were differentiated in the presence of retinoic acid for 4 days. The single cells were obtained after trypsinization. After wash, the cells were incubated with PE-Cy7-labelled EpCAM and PE-labelled CD38 for 30 min on ice. The double positive cells for downstream HiChIP analysis were sorted using FACS.
Instrument	BD Aria II
Software	Flowjo
Cell population abundance	Five independent sorting experiments were performed in this study. For each experiment, approximately 700,000 double positive cells were collected.
Gating strategy	The first step is to use forward and side scatter gating to distinguish populations of cells and to eliminate any debris, dead cells and clumps or doublets. The second step in gating is to gate double-positive cells using a bivariate histogram, which divides the plots in four quadrants for the four possible combinations: double negative, single positive for each antibody, and double positive.

☒ Tick this box to confirm that a figure exemplifying the gating strategy is provided in the Supplementary Information.
